# Utility judgments, risk concerns, and generative AI use intention among first-year university students

**DOI:** 10.3389/fpsyg.2026.1924148

**Published:** 2026-09-21

**Authors:** Chao Yu

**Affiliations:** Department of Psychology, Yichun University, Yichun, China

**Keywords:** first-year university students, generative artificial intelligence, intolerance of uncertainty, output reliability concerns, perceived usefulness, privacy concerns, technology acceptance model, use intention

## Abstract

**Background:**

Generative AI is increasingly embedded in university learning, requiring students to judge its usefulness, how their input data are handled, and whether its outputs are reliable. Adoption research often treats risk as a single broad construct, leaving unclear whether distinct risk concerns relate differently to use intention. This study examined how utility judgments and two distinct risk concerns (privacy and data concerns and output reliability concerns) were associated with generative AI use intention, and whether intolerance of uncertainty was associated with these concerns, among first-year university students.

**Methods:**

Cross-sectional online survey data from 504 first-year students at six public universities in central China were analyzed using confirmatory factor analysis and planned composite-score path modeling. Parallel latent-variable models were estimated to assess sensitivity to score aggregation, measurement error, and estimator choice.

**Results:**

Perceived ease of use was positively associated with perceived usefulness (*β* = 0.507), and perceived usefulness was positively associated with use intention (*β* = 0.602). The indirect association from perceived ease of use to use intention through perceived usefulness was positive (*B* = 0.286, 95% CI [0.210, 0.370]). Intolerance of uncertainty was positively associated with privacy and data concerns and with output reliability concerns, and both associations were stable across composite-score and latent-variable models. Neither concern showed a stable negative association with use intention. In the ordered-categorical analyses, a statistically supported negative coefficient for privacy and data concerns emerged only when output reliability concerns were included simultaneously, whereas the positive coefficient for output reliability concerns received statistical support only in those analyses.

**Conclusion:**

Utility judgments were the most consistent correlates of generative AI use intention, whereas intolerance of uncertainty was consistently associated with stronger risk appraisal. Separating privacy and data concerns from output reliability concerns clarifies that stronger risk concerns do not uniformly correspond to lower use intention.

## 1 Introduction

Generative artificial intelligence (AI) has rapidly become integrated into university students’ study routines. Its interactive capabilities can support conceptual understanding, material organization, written expression, and multiple stages of learning. As students incorporate these tools into their studies, they must judge what information is appropriate to enter, whether generated content is trustworthy, and the extent to which such content can support learning (Kasneci et al., 2023; Miao and Holmes, 2023).

Students’ adoption of generative AI involves both utility evaluation and risk appraisal. Much of the existing adoption literature draws on the Technology Acceptance Model (TAM), which identifies perceived ease of use (PEOU) and perceived usefulness (PU) as central constructs associated with generative AI use intention. In this context, students evaluate whether generative AI is easy to operate and whether it can support learning and improve efficiency; these judgments constitute the utility evaluations associated with use intention (Davis, 1989; Chen et al., 2025).

University students also attend to data privacy and the accuracy of generated content (Maxwell et al., 2025). Existing research, however, often subsumes these issues within an overarching risk construct, giving limited attention to distinct risk sources and their associations with use intention. Privacy and data concerns focus on the handling of personal data or learning materials, while output reliability concerns focus on the accuracy and reliability of generated content. The two may not be associated with use intention in the same way.

Risk concerns may also vary across individuals. Intolerance of uncertainty (IU) is a continuously varying dispositional tendency that reflects sensitivity to unknown situations and their potential consequences (Carleton, 2016). Students with higher IU may attend more closely to unclear potential consequences of generative AI use. Examining IU allows the study to assess whether this disposition is associated with the two risk concerns within a common generative AI-use context.

First-year students are moving from the more guided learning environment of secondary school to more self-directed university study. This transition is often accompanied by changes in resource selection, learning planning, process monitoring, study habits, and patterns of resource use (Liebendörfer et al., 2023; David et al., 2024). The incorporation of generative AI into learning practices makes this transitional period particularly relevant for examining how students evaluate an emerging learning tool.

This study examines the associations of utility judgments and risk concerns with generative AI use intention (BI) among first-year university students. It tests the indirect association between PEOU and BI through PU and the indirect associations between IU and BI through privacy and data concerns (PR) and output reliability concerns (RR). It also examines whether PR and RR show distinct conditional associations with BI. By separating the two risk concerns, the joint framework allows these theory-specified associations to be evaluated while keeping substantively different risk sources distinct. Given the cross-sectional design, their temporal ordering remains unresolved.

## 2 Theoretical analysis and research hypotheses

### 2.1 Utility pathway: perceived ease of use, perceived usefulness, and use intention

As outlined above, TAM posits PEOU and PU as core beliefs associated with technology acceptance. PEOU refers to the degree to which an individual believes that using a particular system requires little effort, whereas PU refers to the degree to which an individual believes that using the system can enhance task performance (Davis, 1989). TAM proposes that judgments of ease of use precede judgments of usefulness: a system that is easy to learn and operate is more likely to be viewed as supporting task completion, improving efficiency, and enhancing outcome quality. Stronger perceptions of usefulness are, in turn, associated with stronger intention to use the system. Research has also reported an indirect association between PEOU and use intention through PU (Venkatesh and Davis, 2000; Lee et al., 2003).

Research with university students is broadly consistent with this utility pathway. Shahzad et al. (2024) reported indirect associations involving PEOU and PU in a Chinese student sample, and Jo (2024) linked favorable evaluations of learning outcomes and task-related benefits with stronger use intention. Findings on the additional direct association between PEOU and use intention remain mixed in generative AI research. Alshammari and Babu (2025) reported a positive conditional association between PU and ChatGPT use intention, whereas PEOU was associated with use intention through satisfaction. Li et al. (2026), by contrast, reported a direct association between PEOU and use intention, together with sequential indirect pathways linking compatibility and popularity to use intention through PEOU and PU. These differences suggest that the role of PEOU beyond its association with PU may depend on the broader model in which the two constructs are examined. On this basis, the present study specifies a theory-guided utility pathway linking PEOU, PU, and BI and proposes the following hypotheses:

*H1*: PEOU is positively associated with PU.

*H2*: PU is positively associated with BI.

*H3*: PEOU is indirectly and positively associated with BI through PU.

### 2.2 Uncertainty and risk pathway: intolerance of uncertainty, risk concerns, and use intention

Intolerance of uncertainty (IU) refers to an individual’s tendency to find it difficult to accept situations in which information is insufficient, outcomes are unpredictable, or the future contains multiple possibilities. Research indicates that individuals with higher IU are more likely to appraise ambiguous or incomplete information as threatening and to attend to possible adverse outcomes and their costs (Carleton, 2016; Milne et al., 2019).

University students using generative AI face uncertainty about how input information is handled and whether generated outputs can be trusted. These uncertainties correspond to the two risk domains examined in this study: PR and RR. Recent adoption studies have linked dimensions of students’ AI literacy, including awareness, evaluative competence, and ethical understanding, with attitudes and perceived behavioral control relevant to use intention (Wang et al., 2025; Zhang et al., 2025). These studies emphasize learned competencies in explaining AI-related attitudes and intentions; the present study complements this work by examining whether the dispositional tendency of IU is associated with both risk domains. Accordingly, the following hypotheses are proposed:

*H4a*: IU is positively associated with PR.

*H4b*: IU is positively associated with RR.

Technology-acceptance and risk-decision research generally treats anticipated adverse consequences as potential deterrents to adoption. When users expect technology use to involve performance failures, information leakage, or other losses, they tend to report lower adoption intention (Featherman and Pavlou, 2003). In the present framework, privacy and data concerns represent risks associated with information handling, whereas output reliability concerns represent risks associated with the reliability of generated outputs. Consistent with this account, research among university students has linked privacy concerns to lower intention to use ChatGPT (Jo, 2024) and broader perceived-risk measures to lower use frequency and use intention (Ortiz-Bonnin and Blahopoulou, 2025).

Recent AI-specific evidence indicates that risk concerns do not always operate as uniform direct correlates of adoption. The revised artificial intelligence device use acceptance (RAIDUA) model positions privacy concerns as a condition shaping the associations among usefulness expectancy, emotional appraisal, and willingness to accept AI-enabled devices (Salam et al., 2026b). Empirical findings are also mixed: Oc et al. (2025) reported a negative association between perceived risk and students’ actual use, whereas Balaskas et al. (2025) reported a positive association between perceived risk and ChatGPT adoption intention. These differences suggest that broad risk measures may combine distinct risk sources and roles. The present study addresses this issue by separating PR and RR and estimating their associations with BI simultaneously. Following the risk-as-deterrent account, both associations are hypothesized to be negative. Accordingly, H5a and H5b provide a direct test of the conventional risk-as-deterrent account. The conditional perspective provides the interpretive framework for departures from these predictions.

*H5a*: PR is negatively associated with BI.

*H5b*: RR is negatively associated with BI.

Taken together, the proposed positive associations of IU with PR and RR and the proposed negative associations of PR and RR with BI imply two negative indirect associations:

*H6a*: IU is indirectly and negatively associated with BI through PR.

*H6b*: IU is indirectly and negatively associated with BI through RR.

### 2.3 Research model and interpretive scope

Figure 1 presents the dual-path research model integrating utility judgments and risk concerns. The utility pathway comprises PEOU, PU, and BI, while the risk pathway comprises IU, PR, RR, and BI. The figure shows the direct paths specified in H1, H2, H4a, H4b, H5a, and H5b and the indirect associations specified in H3, H6a, and H6b.

**Figure 1:**
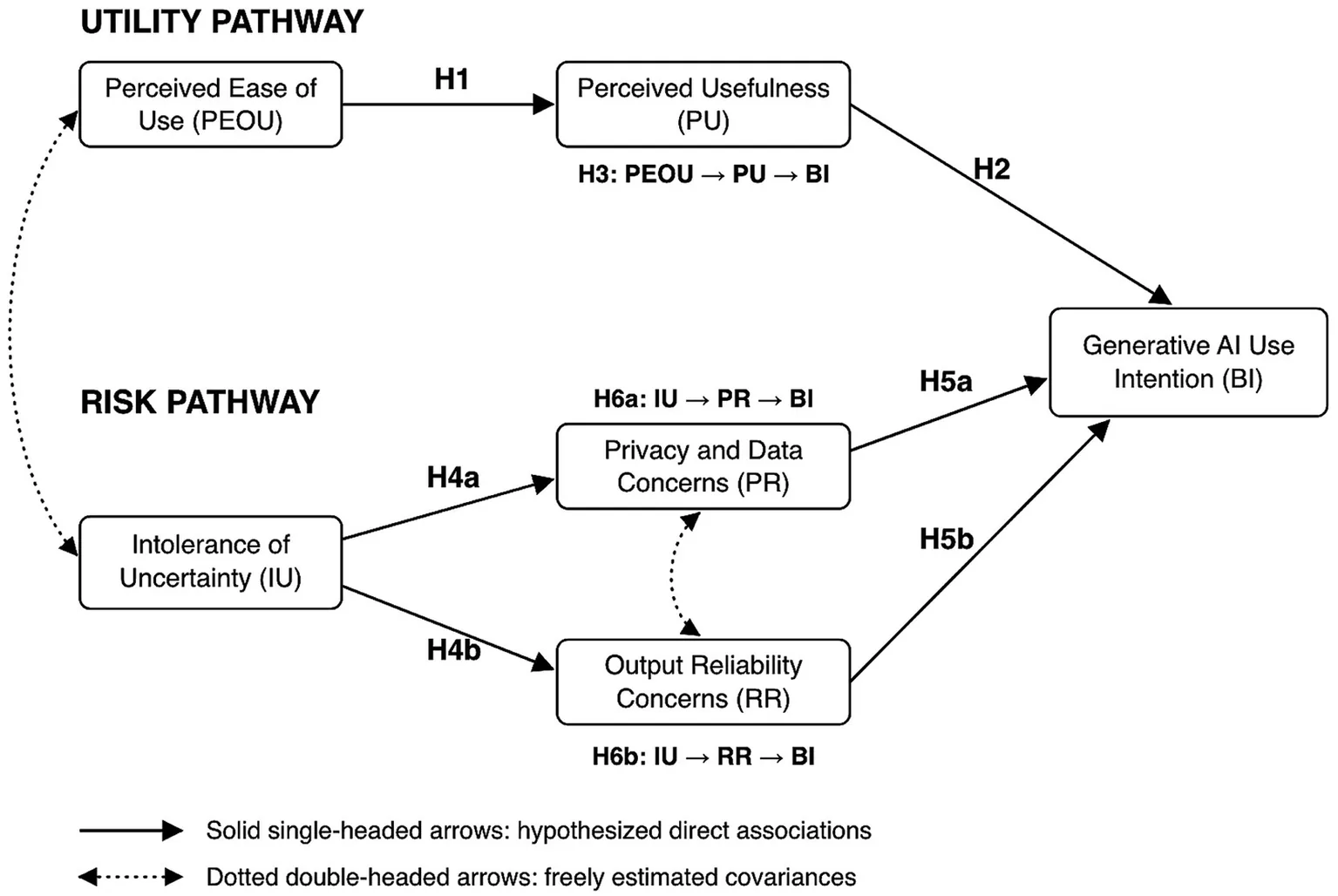
Hypothesized dual-path model (M2a). Solid single-headed arrows represent the hypothesized direct associations corresponding to H1, H2, H4a, H4b, H5a, and H5b. The labels below the model identify the indirect associations specified in H3, H6a, and H6b. Dotted double-headed arrows represent the freely estimated covariance between *IU* and *PEOU* and the residual covariance between *PR* and *RR*; all other endogenous residual covariances were fixed to zero. The exploratory *PEOU* → *BI* and *IU* → *BI* paths are not shown. IU, intolerance of uncertainty; PR, privacy and data concerns; RR, output reliability concerns; PEOU, perceived ease of use; PU, perceived usefulness; BI, generative AI use intention.

Beyond the formal hypotheses, subsequent model comparisons examine whether direct paths from PEOU and IU to BI are warranted in addition to the hypothesized indirect pathways. These paths are treated as exploratory and do not enter the formal hypothesis decisions.

## 3 Methods

### 3.1 Participants and procedure

This study used a cross-sectional online survey of first-year undergraduate students at six public universities in central China. Data were collected from October 19 to December 16, 2025. Counselors responsible for first-year cohorts distributed a link to a questionnaire hosted on Wenjuanxing, a widely used Chinese survey platform, through grade-level WeChat groups. Participation was voluntary, and students read the study information and provided electronic consent before completing the questionnaire. Because the number of eligible students who received the invitation was not recorded, a response rate could not be calculated. The resulting sample was a non-probability, voluntary-response sample. Institutional identifiers were not retained, so responses could not be disaggregated by university.

The cross-sectional design assessed utility judgments, IU, the two risk concerns, and use intention at a single point during students’ transition to university study. IU was measured as a continuously varying disposition (Carleton, 2016), whereas PR and RR were measured as concurrent appraisals. The analyses therefore address contemporaneous associations; temporal ordering and causal direction remain unresolved and are discussed in Section 5.4.

A total of 532 questionnaires were collected. All 31 core measurement items required a response, and no data were missing for these items. Response variability across the 31 items was examined to identify extremely homogeneous patterns that might indicate low response effort, drawing on longstring and within-person response-variability approaches (Meade and Craig, 2012; Dunn et al., 2018). Twenty-eight participants selected the same response option for every core measurement item. These response patterns provided no differentiation across the measured constructs and could distort subsequent estimates; the 28 cases were therefore excluded from the primary analyses.

Following verification of the original questionnaire records and correction of a small number of age entries, the final analytic sample comprised 504 participants: 127 male students (25.2%) and 377 female students (74.8%). The mean age was 19.38 years (*SD* = 1.20; range = 18–29 years). Eligibility was based on first-year university enrollment, and all participants were adults at the time of participation.

### 3.2 Measures

The questionnaire included demographic information, indicators of generative AI use, and 31 core items measuring IU, PR, RR, PEOU, PU, and BI. All core items were rated on a 5-point Likert scale ranging from 1 (“does not describe me at all”) to 5 (“describes me very well”). Construct scores were calculated as the mean of the corresponding items, with higher scores indicating higher levels of each construct. In the narrative text, generative AI use intention is referred to as use intention; BI is retained in the hypotheses, figures, tables, and model notation. Supplementary Table S6 summarizes the construct labels and their analytical representations.

*Intolerance of uncertainty (IU)*. IU was measured using the 12-item short form of the Intolerance of Uncertainty Scale (IUS-12; Carleton et al., 2007). Seven items assessed prospective IU, and five assessed inhibitory IU. Two Chinese–English bilingual researchers with backgrounds in psychology independently translated the English items into Chinese. A third bilingual researcher who had not participated in the initial translation then back-translated the Chinese version. The research team compared the back-translation with the original items and revised the Chinese wording based on this comparison and expert input.

All IUS-12 items were scored in the same direction. The original scale represents overall IU as the sum of the 12 items (Carleton et al., 2007). The primary structural analyses used the mean of the 12 items, which differs from the summed score only by a constant factor. Scores for prospective and inhibitory IU were retained for the supplementary analyses described in Section 3.3.

*Privacy and data concerns*. PR was measured using four items contextualized to generative AI use. The items assessed concerns that personal information, course materials, or assignment content entered into generative AI systems might be collected, stored, misused, or shared, as well as concerns about whether subsequent use of this information could be controlled (Malhotra et al., 2004). PR therefore captures risks related to the handling of input information.

*Output reliability concerns*. RR was measured using four items contextualized to generative AI use. The items assessed concerns about the factual accuracy, verifiability, and limits of applicability of generated content, together with its reliability as a basis for learning or task-related judgments (Ji et al., 2023). RR captures quality-related risks associated with generated outputs.

Both item sets were developed based on their conceptual definitions and contextualized for generative AI use. Experts reviewed the items for content relevance and clarity. The resulting measures were specific to this study rather than directly adopted from a single existing scale. Section 4.1 evaluates their measurement structure, internal consistency, and discriminant validity in relation to adjacent constructs.

*Perceived ease of use, perceived usefulness, and use intention*. The measures of PEOU, PU, and use intention (BI) were developed from the core definitions of TAM and wording used in prior measures, with adaptations for generative AI use (Davis, 1989). The four PEOU items assessed the ease or difficulty of learning to use and operate generative AI. The four PU items assessed its contribution to learning or task efficiency, task-completion outcomes, and task support. The three use-intention items assessed tendencies to continue using generative AI, prioritize its use, and recommend it to others.

*Supplementary use indicators*. The questionnaire also collected indicators of generative AI use, including the number of days used during the previous month, average daily use duration, duration per session, functional breadth, and the extent of use for complex tasks. These indicators were used to describe self-reported use and in exploratory analyses. They were not treated as substitutes for use intention or included in the six-construct measurement model or the main path model.

### 3.3 Data analysis strategy

Confirmatory factor analyses (CFAs) were first conducted on the 31 core measurement items. Because the 5-point Likert responses were treated as ordered categorical variables in the measurement models, the weighted least squares mean- and variance-adjusted (WLSMV) estimator was used (Li, 2016).

Three representations of IU were compared sequentially: a correlated two-factor model comprising prospective and inhibitory IU; the same two-factor model with local residual correlations between items with similar content; and a bifactor model comprising one general factor and two specific factors orthogonal to the general factor and to each other. PR, RR, PEOU, PU, and BI were each examined separately using single-factor models.

An integrated measurement model was then estimated for the six constructs. IU was represented by the bifactor structure, while the other five constructs retained their single-factor specifications. Discriminant validity was examined by comparing this model with three alternatives: one combining PR and RR, one combining PEOU and PU, and one combining both pairs simultaneously.

Model fit was evaluated using χ^2^, the comparative fit index (CFI), Tucker–Lewis index (TLI), root mean square error of approximation (RMSEA) with its 90% confidence interval (CI), and standardized root mean square residual (SRMR). Because the performance of these indices with ordered categorical indicators can vary across estimators, model specifications, and response distributions, they were interpreted together with nested-model comparisons, parameter estimates, and reliability and validity evidence (Savalei, 2021; Xia and Yang, 2019). The mean- and variance-adjusted scaled versions of the fit indices were reported throughout the WLSMV analyses. Nested measurement models were compared using the scaled χ^2^ difference procedure appropriate for WLSMV estimation.

Because all core constructs were assessed by self-report at the same time point, an additional CFA specified all 31 core items as indicators of a single latent factor. This analysis examined whether one common factor could adequately represent the observed item covariance structure.

Cronbach’s α was reported for all constructs. For PR, RR, PEOU, PU, and BI, model-based ω and composite reliability were also reported as indicators of internal consistency, and average variance extracted (AVE) was used to assess convergent validity (Dunn et al., 2014; Fornell and Larcker, 1981). For IU, the standardized loadings on the general factor, explained common variance (ECV), and hierarchical omega (ωH) from the bifactor model were considered together to evaluate whether the 12 items could be interpreted reliably as an overall IU indicator (Rodriguez et al., 2016).

Discriminant validity was initially examined through competing-model comparisons assessing the empirical distinctiveness of PR from RR and PEOU from PU. For the five constructs represented by single-factor models, latent correlations, the heterotrait–monotrait ratio of correlations, version 2 (HTMT2), and the Fornell–Larcker criterion were also examined. IU was excluded from the HTMT2 and Fornell–Larcker matrices because it was represented by a bifactor structure. HTMT2 provided an additional test of construct distinctiveness, while the Fornell–Larcker criterion provided evidence based on AVE (Fornell and Larcker, 1981; Roemer et al., 2021).

The primary hypotheses were tested using composite-score path models estimated with robust maximum likelihood (MLR). Construct scores were calculated by averaging the corresponding items, and the resulting coefficients were expressed on the original score metric. The hypothesized paths and the M0–M3 model-comparison sequence were specified before structural-model estimation, although they were not publicly preregistered. The preceding confirmatory factor and reliability analyses were used to determine whether aggregation of the items into construct scores was psychometrically defensible. Indirect associations were estimated using 5,000 nonparametric bootstrap resamples with percentile-based 95% confidence intervals (CIs; Preacher and Hayes, 2008). In all primary models, IU and PEOU were allowed to covary. The endogenous residual covariance between PR and RR was freely estimated, while all other endogenous residual covariances were fixed to zero unless otherwise specified.

M0 served as the parsimonious baseline model and included paths from PEOU to PU, PU to BI, IU to PR, and IU to RR. M1 added the direct path from PEOU to BI to examine whether it improved the baseline specification. M2a added the paths from PR to BI and RR to BI and served as the formal hypothesis-testing model for H1–H6. M2b extended M2a by adding the path from IU to BI, allowing examination of whether IU retained an association with BI after PR and RR were included. M3 extended M2b by adding the direct path from PEOU to BI, allowing examination of whether PEOU retained an association with BI after the risk pathway had been incorporated. M2b and M3 were treated as exploratory extensions.

Nested structural models were compared using Satorra–Bentler scaled χ^2^ difference tests (Satorra and Bentler, 2010). The Akaike information criterion (AIC) and Bayesian information criterion (BIC) were also reported. For each path model, the results included unstandardized estimates, robust standard errors (SEs), standardized path coefficients, significance levels, explained variance, and fit indices.

To assess whether the substantive conclusions depended on score aggregation, measurement error, or the treatment of five-category items, M2a and M2b were re-estimated as full latent-variable structural equation models using MLR and WLSMV with the items treated as ordered categorical variables. These models retained the structural paths and residual-covariance specifications of their corresponding composite-score models. Results from the composite-score MLR, latent-variable MLR, and latent-variable WLSMV analyses were compared to identify associations that were stable across analytical settings and those that were sensitive to estimator choice.

To examine whether the overall IU results concealed distinct dimension-level patterns, supplementary sensitivity analyses replaced overall IU with prospective IU and inhibitory IU. Each dimension was first entered separately, followed by a model in which both dimensions were entered simultaneously. The resulting estimates were compared with those from the overall-IU model.

Measurement invariance across gender was examined as a supplementary analysis. Because the indicators used five ordered response categories, multigroup WLSMV was selected as the initial estimation method, and response categories were not combined *post hoc*. If the ordinal model did not yield an estimable and admissible solution, the indicators were treated as approximately continuous, and configural, metric, and scalar invariance were examined sequentially using MLR. Changes in CFI, RMSEA, and SRMR were evaluated together with model convergence and parameter admissibility at each level of invariance (Chen, 2007; Putnick and Bornstein, 2016).

The primary analyses used the 504 participants retained after sample screening. To assess whether the screening procedure affected the substantive conclusions, M2a, M2b, and M3 were re-estimated in the full sample of 532 participants using the same model specifications. Age and gender were also added as covariates in the screened sample. Age was mean-centered, and gender was coded as female = 1 and male = 0. Both covariates were included as predictors of PU, PR, RR, and BI.

For the exploratory use indicators described in Section 3.2, Spearman rank correlations were used to examine the associations between use intention and each overall use indicator. Mann–Whitney U tests examined differences in use intention between users and nonusers of specific generative AI functions, with rank-biserial correlations reported as effect sizes. The *p* values from all 15 exploratory tests were adjusted using the Benjamini–Hochberg procedure to control the false discovery rate (FDR).

All confirmatory factor analyses and structural models were estimated in R version 4.6.0 using lavaan version 0.6–21 (Rosseel, 2012).

## 4 Results

### 4.1 Preliminary analyses and measurement models

As shown in Table 1, neither risk concern was negatively correlated with use intention at the zero-order level. The correlation between PR and use intention was not statistically significant, whereas RR showed a weak positive correlation with use intention.

**Table 1:** Descriptive statistics, internal consistency, and interconstruct correlations (*N* = 504).

**Construct**	**k**	***M***	***SD***	**α**	**ω/ωH**
Panel A. Descriptive statistics and internal consistency					
IU	12	3.261	0.600	0.858	0.815^a^
PR	4	3.340	0.824	0.873	0.810
RR	4	3.522	0.715	0.824	0.874
PEOU	4	3.444	0.666	0.758	0.816
PU	4	3.861	0.575	0.856	0.848
BI	3	3.669	0.624	0.752	0.824

Construct	1	2	3	4	5	6
Panel B. Interconstruct correlations.						
1. IU	—					
2. PR	0.300***	—				
3. RR	0.308***	0.575***	—			
4. PEOU	0.173***	0.116**	0.226***	—		
5. PU	0.153***	0.146***	0.218***	0.507***	—	
6. BI	0.231***	0.051	0.129**	0.337***	0.600***	—

k = number of items. Composite scores were computed as the mean of the respective items. Panel B reports Pearson correlations. All items were coded from 1 = does not describe me at all to 5 = describes me very well.

a IU reports hierarchical omega (ωH) for the general factor from the bifactor model. **p < 0.01. ***p < 0.001.

Table 2 summarizes the fit statistics for the three candidate representations of the IUS-12. Among the three candidate representations of the IUS-12, the orthogonal bifactor model showed the strongest relative fit, χ^2^(42) = 228.374, CFI = 0.963, TLI = 0.942, RMSEA = 0.094, 90% CI [0.082, 0.106], and SRMR = 0.043. The fit indices were mixed: CFI and SRMR were comparatively favorable, while the elevated RMSEA indicated limited absolute fit.

**Table 2:** Comparison of measurement models for intolerance of uncertainty (*N* = 504).

Model	χ^2^(*df*)	CFI	TLI	RMSEA [90% CI]	SRMR	Conclusion
Correlated two-factor model	490.615 (53)	0.913	0.892	0.128 [0.118, 0.139]	0.071	Limited baseline fit
Correlated two-factor model, allowing iu2 ~~ iu7	360.583 (52)	0.939	0.922	0.109 [0.098, 0.119]	0.059	Improved, but still limited fit
Orthogonal bifactor model	228.374 (42)	0.963	0.942	0.094 [0.082, 0.106]	0.043	Best fit of the three models

All models were estimated using WLSMV. χ^2^, CFI, TLI, and RMSEA are WLSMV-scaled fit statistics; SRMR is the standardized root mean square residual. All models converged and passed post-estimation checks; all χ^2^ tests were *p* < 0.001.

In the second model, the iu2 ~~ iu7 residual covariance was included solely to examine local dependence in the correlated two-factor model and was not retained in the final six-construct integrated measurement model. The bifactor model comprises one general intolerance-of-uncertainty factor and two orthogonal specific factors (prospective and inhibitory).

The bifactor-specific indices informed the scoring decision. The general IU factor accounted for a substantial proportion of the common variance (ECV = 0.753), and the total score predominantly reflected reliable variance attributable to the general factor (ωH = 0.815). After the general factor was controlled, the prospective and inhibitory specific factors retained little unique reliable variance, as indicated by hierarchical omega estimates for the specific factors (ωHS = 0.068 and 0.188, respectively). These results supported the use of the 12-item overall IU score in the primary structural model. Dimension-level sensitivity results are reported in Section 4.4.2.

The final integrated measurement model was estimated using WLSMV. PR, RR, PEOU, PU, and BI were each represented by a single factor, while IU retained the bifactor structure comprising one general factor and two orthogonal specific factors. Residual correlations between pr1 and pr2 and between pu3 and pu4 were retained because of similarities in item content. The model showed acceptable fit across the reported WLSMV-scaled indices: χ^2^(405) = 1107.362, CFI = 0.958, TLI = 0.951, RMSEA = 0.059, 90% CI [0.055, 0.063], and SRMR = 0.063 (Table 3).

**Table 3:** Fit of the final integrated measurement model and construct validity assessment (*N* = 504).

Model	χ^2^(*df*)	CFI	TLI	RMSEA [90% CI]	SRMR
Panel A. Fit of the final and competing models					
Final six-construct model	1107.362 (405)	0.958	0.951	0.059 [0.055, 0.063]	0.063

Model	χ^2^(*df*)	CFI	TLI	RMSEA [90% CI]	SRMR	ΔCFI
*Absolute fit of competing models*						
PR/RR-combined model	1363.374 (410)	0.942	0.935	0.068 [0.064, 0.072]	0.068	−0.015
PEOU/PU-combined model	1307.804 (410)	0.946	0.938	0.066 [0.062, 0.070]	0.068	−0.012
Both-pairs-combined model	1546.123 (414)	0.932	0.923	0.074 [0.070, 0.078]	0.073	−0.026

Comparison	Scaled Δχ^2^ (Δ*df*)	*p*
*Nested comparisons of competing models.*		
PR/RR-combined model vs. final six-construct model	173.240 (5)	< 0.001
PEOU/PU-combined model vs. final six-construct model	107.340 (5)	< 0.001
Both-pairs-combined model vs. final six-construct model	249.350 (9)	< 0.001

Construct	Range of Standardized Factor Loadings	Model ω / ωH	CR	AVE	ECV
Panel B. Standardized factor loadings and convergent validity					
General intolerance of uncertainty (IU)	0.452–0.758	0.815ᵃ	—	—	0.753
Privacy and data concerns (PR)	0.711–0.900	0.810	0.898	0.690	—
Output reliability concerns (RR)	0.718–0.855	0.874	0.874	0.635	—
Perceived ease of use (PEOU)	0.590–0.828	0.816	0.816	0.531	—
Perceived usefulness (PU)	0.792–0.874	0.848	0.913	0.725	—
Generative AI use intention (BI)	0.711–0.839	0.824	0.824	0.610	—

All models in Panel A were estimated using WLSMV. χ^2^, CFI, TLI, and RMSEA are WLSMV-scaled fit statistics; SRMR is the standardized root mean square residual. ΔCFI values were calculated from unrounded CFI estimates and describe deterioration in absolute fit relative to the final six-construct model. Scaled Δχ^2^ is the nested difference test comparing each competing model with the final six-construct model. All models retained the same orthogonal bifactor specification for IU and the same local residual covariances (pr1 ~~ pr2 and pu3 ~~ pu4).

For IU in Panel B, ωH and ECV pertain to the general factor in the bifactor model. The final measurement models for PR and PU retained local residual covariances between pr1 and pr2 and between pu3 and pu4, respectively. Model ω was computed retaining those covariance structures. CR was calculated from standardized factor loadings and residual variances under the conventional formula and did not incorporate the local residual covariances. Accordingly, model ω and CR coincide for RR, PEOU, and BI but differ for PR and PU; both indices, together with AVE, are reported for all five single-factor constructs. ^a^ IU reports hierarchical omega (ωH) for the general factor from the bifactor model.

The single-factor model comprising all 31 core measurement items converged but fit the data poorly: χ^2^(434) = 7369.909, CFI = 0.581, TLI = 0.551, RMSEA = 0.178, 90% CI [0.175, 0.182], and SRMR = 0.190. Its fit was substantially poorer than that of the final integrated measurement model. The poor fit of the single-factor model indicated that one common factor was insufficient to account for the observed covariance structure, but it did not rule out common-method influence arising from other sources (Podsakoff et al., 2024).

The competing-model comparisons supported the empirical distinctiveness of the two pairs of adjacent constructs. Relative to the final integrated measurement model, combining PR and RR reduced CFI from 0.958 to 0.942 and increased RMSEA from 0.059 to 0.068, scaled Δχ^2^(5) = 173.240, *p* < 0.001. Combining PEOU and PU reduced CFI to 0.946 and increased RMSEA to 0.066, scaled Δχ^2^(5) = 107.340, *p* < 0.001. Simultaneously combining both pairs produced a further deterioration in model fit, CFI = 0.932, RMSEA = 0.074, scaled Δχ^2^(9) = 249.350, *p* < 0.001. These comparisons supported treating PR and RR, and PEOU and PU, as empirically distinct constructs.

For the five constructs represented by single-factor models, standardized factor loadings ranged from 0.590 to 0.900, composite reliability ranged from 0.816 to 0.913, and AVE ranged from 0.531 to 0.725. The additional discriminant-validity assessments were consistent with the competing-model results. All HTMT2 values were below 0.85, and the square root of AVE for each construct exceeded the absolute value of its latent correlations with the other four constructs. The strongest latent correlation was observed between PU and BI (φ = 0.767, HTMT2 = 0.755), followed by the correlation between PR and RR (φ = 0.733, HTMT2 = 0.701). In both cases, the square roots of AVE remained greater than the corresponding interconstruct correlation. The complete HTMT2 and Fornell–Larcker matrices are presented in Supplementary Tables S1, S2.

### 4.2 Structural path models and model comparisons

Following the item-level measurement assessment, construct mean scores were used in the primary composite-score path models estimated with MLR, as described in Section 3.3. Table 4 reports model fit, the planned nested comparisons, and explained variance for M0, M1, M2a, M2b, and M3. The latent-variable structural models, IU dimension analyses, and gender measurement-invariance analyses are reported as supplementary sensitivity analyses in Section 4.4 and Supplementary Tables S3–S5.

**Table 4:** Fit, comparisons, and explained variance of structural path models (*N* = 504).

Model	Newly freed paths	χ^2^(*df*)	CFI	TLI	RMSEA [90% CI]	SRMR	AIC/BIC	BI R^2^
Panel A. Model fit and explained variance in use intention								
M0	—	38.23 (9)	0.933	0.889	0.080 [0.058, 0.104]	0.071	5464.37/5540.37	0.360
M1	PEOU→BI	37.03 (8)	0.934	0.876	0.085 [0.061, 0.110]	0.070	5465.28/5545.50	0.362
M2a	PR → BI; RR → BI	36.69 (7)	0.932	0.855	0.092 [0.067, 0.118]	0.073	5466.87/5551.32	0.363
M2b	IU → BI*	21.43 (6)	0.965	0.912	0.071 [0.044, 0.101]	0.063	5449.37/5538.04	0.386
M3	PEOU → BI	20.56 (5)	0.964	0.893	0.079 [0.049, 0.110]	0.063	5450.92/5543.81	0.387

Comparison	Scaled Δχ^2^ (Δ*df*)	*p*
Panel B. Nested model comparisons		
M1 vs. M0: PEOU → BI	0.912 (1)	0.340
M2a vs. M0: PR → BI, RR → BI	1.180 (2)	0.554
M2b vs. M2a: IU → BI*	12.015 (1)	< 0.001
M3 vs. M2b: PEOU → BI	0.402 (1)	0.526

Endogenous variable	M2a (hypothesized dual-path model)	M2b (exploratory extension)
Panel C. Explained variance in endogenous variables (R^2^)		
Perceived usefulness (PU)	0.257	0.257
Privacy and data concerns (PR)	0.090	0.090
Output reliability concerns (RR)	0.095	0.095
Generative AI use intention (BI)	0.363	0.386

All models were estimated using MLR; IU and PEOU were allowed to covary, only the PR–RR endogenous residual covariance was freely estimated, and all other endogenous residual covariances were fixed at zero. M0 is the baseline model, including PEOU → PU → BI, IU → PR, and IU → RR; newly freed paths in subsequent models are shown in the “Newly Freed Paths” column. χ^2^, CFI, TLI, and RMSEA are MLR robust statistics. Nested comparisons used the Satorra–Bentler scaled χ^2^ difference test. Lower AIC and BIC values indicate a better relative information-criterion fit. * Exploratory path.

The parsimonious baseline model, M0, yielded χ^2^(9) = 38.23, CFI = 0.933, TLI = 0.889, RMSEA = 0.080, 90% CI [0.058, 0.104], SRMR = 0.071, AIC = 5464.37, and BIC = 5540.37. Adding the direct path from PEOU to BI in M1 did not improve model fit, Satorra–Bentler scaled Δχ^2^(1) = 0.912, *p* = 0.340, and the added path was not statistically significant, *p* = 0.318. M2a added the paths from PR and RR to BI. Its fit did not differ significantly from that of M0, Satorra–Bentler scaled Δχ^2^(2) = 1.180, *p* = 0.554, and its AIC and BIC were not lower than those of the baseline model.

M2b extended M2a by adding the path from IU to BI and showed a significant improvement in fit, Satorra–Bentler scaled Δχ^2^(1) = 12.015, *p* < 0.001. The model yielded χ^2^(6) = 21.43, CFI = 0.965, TLI = 0.912, RMSEA = 0.071, 90% CI [0.044, 0.101], SRMR = 0.063, AIC = 5449.37, and BIC = 5538.04. The proportion of variance explained in BI increased from 36.3% in M2a to 38.6% in M2b. Adding the direct path from PEOU to BI in M3 produced no further improvement, Satorra–Bentler scaled Δχ^2^(1) = 0.402, *p* = 0.526, and the added path remained nonsignificant, *p* = 0.518.

The comparison between M2a and M0 evaluated the joint contribution of the two paths from PR and RR to BI. Formal decisions regarding H5a–H6b were based on the corresponding path estimates and bootstrap confidence intervals in M2a, as specified in Section 3.3. The exploratory IU-to-BI association in M2b is reported in Section 4.3.

Absolute fit indices varied across the five composite-score models. Given their small degrees of freedom, the indices were mixed: RMSEA ranged from 0.071 to 0.092, TLI from 0.855 to 0.912, and SRMR from 0.063 to 0.073. M2b showed the most favorable absolute fit, whereas the indices for M2a were comparatively weaker. These values are presented in Table 4 and were interpreted together with the planned nested-model comparisons.

### 4.3 Hypothesized path tests and exploratory extension

Table 5 reports the path estimates and indirect associations for the formal hypothesis-testing model, M2a. Within the utility pathway, PEOU was positively associated with PU, *B* = 0.438, robust *SE* = 0.047, *z* = 9.344, *p* < 0.001, *β* = 0.507, and PU was positively associated with use intention, *B* = 0.653, robust *SE* = 0.050, *z* = 13.021, *p* < 0.001, *β* = 0.602. The bootstrap analysis also showed a positive indirect association between PEOU and use intention through PU, *B* = 0.286, bootstrap *SE* = 0.041, 95% CI [0.210, 0.370]. H1, H2, and H3 were supported.

**Table 5:** Path estimates and indirect associations in the hypothesized dual-path model (M2a; *N* = 504).

Hyp.	Path	*B*	Robust *SE*	*z*	*p*	*β*	Conclusion
Panel A. Hypothesized direct paths							
H1	PEOU → PU	0.438	0.047	9.344	< 0.001	0.507	Supported
H2	PU → BI	0.653	0.050	13.021	< 0.001	0.602	Supported
H4a	IU → PR	0.412	0.068	6.042	< 0.001	0.300	Supported
H4b	IU → RR	0.367	0.061	6.017	< 0.001	0.308	Supported
H5a	PR → BI	−0.040	0.033	−1.206	0.228	−0.053	Not supported
H5b	RR → BI	0.025	0.044	0.555	0.579	0.028	Not supported

Hyp.	Indirect path	*B*	Bootstrap *SE*	95% CI	Conclusion
Panel B. Bootstrap tests of hypothesized indirect associations					
H3	PEOU→PU → BI	0.286	0.041	[0.210, 0.370]	Supported
H6a	IU → PR → BI	−0.017	0.014	[−0.043, 0.011]	Not supported
H6b	IU → RR → BI	0.009	0.017	[−0.022, 0.045]	Not supported

M2a is the formal hypothesis-testing model and adds PR → BI and RR → BI to M0. Panel A reports MLR estimates; B is the unstandardized path coefficient, SE is the robust standard error, and *β* is the standardized path coefficient. Panel B reports unstandardized indirect associations from 5,000 nonparametric bootstrap resamples, with percentile 95% confidence intervals. Support for H1–H6 was determined from the corresponding direct path or indirect association in M2a. IU, intolerance of uncertainty; PR, privacy and data concerns; RR, output reliability concerns; PEOU, perceived ease of use; PU, perceived usefulness; BI, generative AI use intention.

Within the risk pathway, IU was positively associated with both PR, *B* = 0.412, robust *SE* = 0.068, *z* = 6.042, *p* < 0.001, *β* = 0.300, and RR, *B* = 0.367, robust *SE* = 0.061, *z* = 6.017, *p* < 0.001, *β* = 0.308. H4a and H4b were supported.

In the formal hypothesis-testing model, M2a, neither risk concern showed the hypothesized negative conditional association with use intention. The coefficient for PR was *B* = −0.040, robust *SE* = 0.033, *z* = −1.206, *p* = 0.228, *β* = −0.053, and the coefficient for RR was *B* = 0.025, robust *SE* = 0.044, *z* = 0.555, *p* = 0.579, *β* = 0.028. H5a and H5b were not supported.

The percentile-bootstrap indirect association through PR was *B* = −0.017, 95% CI [−0.043, 0.011], and the corresponding indirect association through RR was *B* = 0.009, 95% CI [−0.022, 0.045]. The combined indirect association through the two risk concerns was *B* = −0.008, 95% CI [−0.035, 0.025]. Because the confidence intervals for both hypothesized indirect associations included zero, H6a and H6b were not supported.

In the exploratory M2b model, the added path from IU to use intention was positive, *B* = 0.173, robust *SE* = 0.053, *z* = 3.283, *p* = 0.001, *β* = 0.166. After the direct path from PEOU to BI was added in M3, the IU-to-BI path remained statistically significant, *B* = 0.171, robust *SE* = 0.054, *z* = 3.165, *p* = 0.002, *β* = 0.164, while the PEOU-to-BI path remained nonsignificant. The IU-to-use-intention path was exploratory and did not enter the formal hypothesis decisions. Its stability across latent-variable estimators is examined in Section 4.4.1.

### 4.4 Robustness and exploratory analyses

#### 4.4.1 Latent-variable and estimator sensitivity

The latent-variable MLR version of M2a showed acceptable fit, scaled χ^2^(412) = 630.907, robust CFI = 0.953, robust TLI = 0.947, robust RMSEA = 0.037, and SRMR = 0.065. The results reproduced the stable components of the primary composite-score model. PEOU was positively associated with PU (*β* = 0.645, *p* < 0.001), PU was positively associated with use intention (*β* = 0.759, *p* < 0.001), and the standardized indirect association through PU was positive (*β* = 0.490, *p* < 0.001). General IU was positively associated with PR (*β* = 0.309, *p* < 0.001) and RR (*β* = 0.340, *p* < 0.001). The conditional coefficients from PR (*β* = −0.110, *p* = 0.119) and RR (*β* = 0.085, *p* = 0.281) to use intention were not statistically significant.

The ordered-item WLSMV version of M2a yielded the following scaled fit indices: χ^2^(412) = 1250.898, CFI = 0.949, TLI = 0.943, RMSEA = 0.064, 90% CI [0.060, 0.068], and SRMR = 0.074. The utility pathway and the positive associations of general IU with both risk concerns were again reproduced. In the joint-risk model, however, PR had a negative conditional coefficient for use intention (*β* = −0.196, *p* = 0.002), while RR had a positive conditional coefficient (*β* = 0.289, *p* < 0.001).

Diagnostic WLSMV models clarified these coefficients. When the RR-to-BI path was omitted, the PR coefficient was positive and nonsignificant (*β* = 0.047, *p* = 0.166). When the PR-to-BI path was omitted, RR retained a smaller positive coefficient (*β* = 0.147, *p* < 0.001). The residual correlation between PR and RR in the joint model was 0.686. These results indicate that the negative PR coefficient emerged only when RR was included simultaneously, whereas statistical support for the positive RR coefficient was specific to the ordered-item WLSMV analyses.

The standardized indirect association through PR was negative under WLSMV (*β* = −0.074, *p* = 0.002), the indirect association through RR was positive (*β* = 0.131, *p* < 0.001), and their combined indirect association was also positive (*β* = 0.057, *p* = 0.001). The corresponding indirect associations were not statistically significant in the latent-variable MLR model. The estimator-dependent risk coefficients and indirect associations therefore did not alter the formal decisions for H5a–H6b.

The exploratory association between IU and use intention was also sensitive to the analytical setting. In the latent-variable MLR version of M2b, the coefficient was positive (*β* = 0.183, *p* = 0.004), although the Satorra–Bentler scaled χ^2^ difference test comparing M2b with M2a was not statistically significant (*p* = 0.089). Under WLSMV, the coefficient was *β* = 0.100 (*p* = 0.058), and the corresponding scaled χ^2^ difference test was also nonsignificant (*p* = 0.070). Complete parallel results are presented in Supplementary Table S3.

#### 4.4.2 IU dimension sensitivity

When entered separately, prospective IU was positively associated with PR (*β* = 0.269, *p* < 0.001) and RR (*β* = 0.281, *p* < 0.001). Inhibitory IU showed similar positive associations with PR (*β* = 0.280, *p* < 0.001) and RR (*β* = 0.282, *p* < 0.001). None of the corresponding indirect associations with use intention was statistically significant, and all percentile-based 95% confidence intervals from 5,000 nonparametric bootstrap resamples included zero.

When both dimensions were entered simultaneously, prospective and inhibitory IU each remained positively associated with both risk concerns, while neither showed a statistically significant indirect association with use intention. In the exploratory direct-path models, prospective IU (*β* = 0.145, *p* = 0.002) and inhibitory IU (*β* = 0.153, *p* = 0.004) were each positively associated with use intention when examined separately. After both dimensions were included in the same model, neither dimension-specific coefficient was statistically significant: prospective IU, *β* = 0.078, *p* = 0.109; inhibitory IU, *β* = 0.104, *p* = 0.085. The analyses therefore provided no evidence of a distinct prospective- or inhibitory-IU pathway and did not alter the conclusions based on overall IU. Complete estimates are presented in Supplementary Table S4.

#### 4.4.3 Gender measurement-invariance sensitivity

The ordinal multigroup WLSMV configural model could not be estimated because six item-by-gender combinations contained an empty response category, and several additional cells in the lowest response category contained fewer than five observations. Sparse categories were not merged *post hoc* because doing so would alter the original response scale and introduce data-dependent scoring decisions.

As a supplementary analysis, the five-category indicators were treated as approximately continuous and estimated using multigroup MLR. The configural model showed acceptable fit, robust CFI = 0.934, robust TLI = 0.924, robust RMSEA = 0.046, and SRMR = 0.059. The metric-invariance model also yielded an admissible solution, robust CFI = 0.930, robust TLI = 0.923, robust RMSEA = 0.046, and SRMR = 0.063. Relative to the configural model, the changes were ΔCFI = −0.004, ΔRMSEA < 0.001, and ΔSRMR = 0.004. The scalar model produced a negative residual variance for iu2 in the smaller male group and therefore failed the post-estimation admissibility check.

The supplementary continuous-indicator analysis provided evidence consistent with configural and metric invariance, but scalar invariance could not be established. Latent-mean and structural comparisons across gender were therefore not conducted. Fit statistics and change indices for each invariance level are reported in Supplementary Table S5.

#### 4.4.4 Sample and covariate robustness

The model-comparison pattern in the full sample of 532 participants closely matched that in the screened sample. Adding the PEOU-to-BI path to M0 did not improve fit, Satorra–Bentler scaled Δχ^2^(1) = 1.913, *p* = 0.167. Adding the PR-to-BI and RR-to-BI paths jointly also produced no significant improvement over M0, scaled Δχ^2^(2) = 0.875, *p* = 0.646. The exploratory addition of the IU-to-BI path to M2a improved fit, scaled Δχ^2^(1) = 15.127, *p* < 0.001, whereas adding the PEOU-to-BI path to M2b produced no further improvement, scaled Δχ^2^(1) = 0.719, *p* = 0.396.

The directions and significance patterns of the core paths in M2a were also consistent across the two samples. Using 5,000 nonparametric percentile-bootstrap resamples, the indirect association between PEOU and BI through PU remained positive in the full sample, *B* = 0.324, 95% CI [0.249, 0.407]. Neither of the two specific indirect associations from IU to BI through PR or RR, nor their combined indirect association, was statistically significant. In M2b, the total association between IU and BI, comprising the exploratory direct path and the two risk-related indirect associations, was positive, *B* = 0.164, 95% CI [0.086, 0.252]. Across the screened and full samples, absolute differences in the standardized coefficients for the six hypothesized direct paths and the exploratory IU-to-BI path did not exceed 0.055. Excluding the 28 participants who selected the same response option for all core items therefore had little effect on the estimated associations.

This pooled composite-score analysis served solely as a robustness check; latent-mean comparisons were outside its scope. Adjustment for age and gender in the screened sample produced similarly stable results. Absolute changes in the core standardized path estimates did not exceed 0.007. The variance explained in BI increased from 36.3 to 36.7% in M2a and from 38.6 to 39.1% in M2b. The exploratory IU-to-BI path remained statistically significant, and M2b continued to fit significantly better than M2a, Satorra–Bentler scaled Δχ^2^(1) = 12.098, *p* < 0.001. Overall, neither sample screening nor adjustment for age and gender materially altered the main pattern of associations.

#### 4.4.5 Associations with self-reported use indicators

Panel A of Table 6 shows that, after FDR adjustment, use intention was positively correlated with Days used, Time per session, and Functional breadth. The correlations with Daily use time and Complex-task use were not statistically significant.

**Table 6:** Exploratory associations between use indicators and generative AI use intention (*N* = 504).

Use indicator	Spearman *r*_s	Raw *p*	FDR-adjusted *p*
Panel A. Rank correlations between overall use indicators and use intention			
Days used (Q35)	0.242	< 0.001	< 0.001
Daily use time (Q36)	−0.006	0.900	0.900
Time per session (Q37)	0.164	< 0.001	< 0.001
Functional breadth (Q38)	0.235	< 0.001	< 0.001
Complex-task use (Q39)	−0.029	0.510	0.696

Function	Users n (%)	Users BI *M*	Nonusers BI *M*	Rank-biserial *r*	Raw *p*	FDR-adjusted *p*
Panel B. Between-group comparisons in use intention by use of specific functions						
Summarization or search	351 (69.6)	3.685	3.634	0.022	0.683	0.731
Translation	347 (68.8)	3.720	3.558	0.167	0.002	0.005
Data analysis	273 (54.2)	3.719	3.610	0.099	0.046	0.070
Image generation and editing	232 (46.0)	3.671	3.668	0.022	0.661	0.731
Writing/document reading	285 (56.5)	3.788	3.514	0.254	< 0.001	< 0.001
Academic writing	171 (33.9)	3.764	3.621	0.136	0.010	0.016
Slide generation	238 (47.2)	3.744	3.603	0.136	0.006	0.013
Coursework completion	306 (60.7)	3.764	3.524	0.216	< 0.001	< 0.001
Document-based chat	107 (21.2)	3.804	3.633	0.158	0.009	0.016
Code generation/coding assistance	33 (6.5)	3.657	3.670	−0.056	0.577	0.722

Panel A reports two-sided Spearman rank correlations between BI and the use indicators. Panel B reports two-sided Mann–Whitney comparisons between users and nonusers of each function. Positive rank-biserial correlations indicate higher BI among users. The Q38 function items and functional breadth were recoded from the original multiple-response text. FDR p values were adjusted across the 15 exploratory tests (five in Panel A and 10 in Panel B) using the Benjamini–Hochberg procedure. All results are exploratory cross-sectional associations. BI = generative AI use intention.

Function-specific comparisons showed higher use intention among users than nonusers for Writing/document reading (rank-biserial correlation = 0.254, FDR-adjusted *p* < 0.001), Coursework completion (0.216, *p* < 0.001), Translation (0.167, *p* = 0.005), Document-based chat (0.158, *p* = 0.016), Slide generation (0.136, *p* = 0.013), and Academic writing (0.136, *p* = 0.016). The unadjusted comparison for Data analysis was statistically significant (*p* = 0.046), but did not remain so after the Benjamini–Hochberg adjustment (FDR-adjusted *p* = 0.070). No statistically significant differences were found for Summarization or search, Image generation and editing, or Code generation/coding assistance.

## 5 Discussion

### 5.1 Main findings

The most consistent findings were the utility pathway and the associations of IU with the two risk concerns. Across the primary composite-score model and both latent-variable analyses, PEOU was positively associated with PU, PU was positively associated with use intention, and IU was positively associated with both PR and RR. The associations of the two risk concerns with use intention were considerably less stable. PR was unrelated to use intention at the zero-order level, while RR showed a weak positive correlation; their conditional coefficients subsequently varied with joint adjustment and estimator choice. The analyses therefore provided no consistent evidence that either risk concern was associated with lower use intention. Exploratory analyses also linked use intention with several aspects of self-reported use, including days used, time per session, functional breadth, and the use of several specific functions. In the primary composite-score model, the standardized coefficients for the two utility paths were 0.507 and 0.602, whereas the conditional coefficients from PR and RR to use intention had absolute values no greater than 0.053.

Evidence for the exploratory IU–use intention association weakened when measurement error and IU dimensionality were taken into account: a positive coefficient emerged in the composite-score model, but support diminished in the latent-variable model comparisons and when prospective and inhibitory IU were entered simultaneously. This pattern points to variance shared by the two IU dimensions rather than a dimension-specific association. The cross-sectional analyses cannot determine the process underlying this result. One possibility is that stronger uncertainty-related concern can coexist with greater motivation to use generative AI, an interpretation considered in Section 5.2.

### 5.2 Theoretical implications

The findings refine the application of TAM to generative AI in learning contexts. Across composite-score and latent-variable analyses, PEOU was associated with use intention through PU, indicating that students’ judgments about whether generative AI can support learning and task completion form the more proximal utility basis of use intention. The consistency of this pathway across scoring and estimation approaches strengthens the interpretation of PU as the central link between ease of use and use intention in this context.

The risk findings point to a more conditional account. PR and RR were empirically distinguishable, yet neither showed a stable association with use intention across analytical settings. Their coefficients changed when the two concerns were estimated jointly and when the treatment of the item responses changed. RAIDUA offers a relevant explanation by modeling privacy concerns as a moderator of the association between usefulness expectancy and emotional appraisal within AI acceptance (Salam et al., 2026b). Applied to the present framework, the relevance of PR and RR for use intention may vary with perceived usefulness, trust, task stakes, verification capacity, and institutional safeguards.

This interpretation also helps situate the divergent findings reported in earlier AI-acceptance research. Perceived risk has been associated with lower student use in some studies and with stronger adoption intention in others, while related models have incorporated trust and academic-integrity concerns alongside anticipated benefits (Jo, 2024; Shahzad et al., 2024; Oc et al., 2025; Balaskas et al., 2025; Ortiz-Bonnin and Blahopoulou, 2025). Broad risk measures may therefore combine concerns that have different relationships with use intention. Separating input-related privacy and data concerns from output-related reliability concerns provides a more differentiated basis for examining those relationships. The present results also suggest that their conditional coefficients can depend on how overlapping concerns are represented in the model.

The IU findings identify a different part of the framework. General IU was consistently associated with both PR and RR, and the dimension-level analyses did not reveal a distinct prospective- or inhibitory-IU pathway. Together with the limited unique reliable variance retained by the two specific dimensions, this pattern suggests that overall IU captures a broad sensitivity to uncertain consequences across both input-data handling and generated-output reliability. Risk sensitivity and the association of risk appraisals with use intention should therefore be treated as separate theoretical questions: IU was consistently related to stronger risk appraisal, while the subsequent associations of those appraisals with use intention varied across models.

The positive exploratory association between IU and use intention raises the possibility that uncertainty-related concern can coexist with motivation to use generative AI. Students may turn to generative AI to seek information, clarification, or reassurance when facing uncertain learning tasks. The weakening of this association in the latent-variable comparisons and after simultaneous adjustment for the two IU dimensions indicates that it should be interpreted as a preliminary shared-variance pattern. Research that directly measures information seeking, reassurance seeking, avoidance, trust calibration, and task-specific reliance is needed to distinguish the processes that may underlie it.

### 5.3 Practical implications

The stability of the utility pathway identifies a clear implementation priority. Universities and instructors can use curriculum-embedded demonstrations to show how generative AI supports specific learning tasks, reduce avoidable interaction burdens, and clarify the boundaries of appropriate use. Because PEOU was associated with use intention through PU, implementation efforts should help students evaluate concrete learning gains and task fit.

In this sample, stronger risk concerns did not consistently correspond to lower use intention. General concern may therefore have limited relevance to students’ intentions unless it is connected to clear rules, task-specific guidance, and opportunities to evaluate the consequences of AI use. Universities can support this process by combining institutional safeguards with instruction that develops students’ capacity to make accountable decisions about generative AI.

The distinction between the two risk domains calls for different forms of support. For output reliability, universities should evaluate the suitability of generative AI tools for particular learning tasks, communicate known limitations and potential biases, and specify when students must verify sources, compare outputs with external evidence, or seek human review. These measures make the limits of AI-generated content and the requirements for its responsible use more transparent.

For privacy and data handling, institutional policies should identify approved systems, restricted categories of information, and the applicable conditions for data retention and sharing. Clear requirements are also needed for disclosing AI involvement, maintaining academic integrity, and using generative AI in high-stakes tasks. Together, these arrangements can support calibrated trust by linking confidence in AI use to available evidence, task consequences, and institutional protections.

Instructors have a complementary role. They should identify the judgments that students are expected to make independently and design assessments that make reasoning, verification, and revision visible. The Integrated Collaborative Reflection Model (ICRM; Salam et al., 2026a) offers a practical structure for this purpose. Students can complete an AI-supported task, record an individual evaluation of the output, compare evidence and concerns through collaborative reflection, formulate recommendations, and reach a justified decision about whether the output should be accepted, revised, disclosed, or rejected. This sequence can help convert general concerns into explicit criteria for responsible use. Policy support can extend these practices through minimum standards for academic integrity, equitable access to AI-literacy education, and additional assistance for students with limited prior experience.

### 5.4 Limitations and future research

The cross-sectional, single-source design limits the temporal and causal interpretation of the observed associations. The poor fit of the single-factor CFA showed that one common latent factor was insufficient to reproduce the observed covariance structure. Shared item formats, simultaneous measurement, response tendencies, and the survey context may nevertheless have contributed method-related covariance (Podsakoff et al., 2024). Longitudinal, experimental, multi-source, and task-based designs are needed to establish temporal ordering and distinguish substantive associations from shared-method covariance.

The measurement findings also require independent replication. The bifactor IUS-12 model and the composite-score M2a model produced mixed fit indices. Within this sample, ECV, ωH, the dimension-level sensitivity analyses, and the parallel latent-variable models supported the use of the overall IU score and the resulting interpretation. PR and RR were assessed using study-specific, contextualized indicators, and their temporal stability, predictive validity, and associations with observed risk behavior remain unknown. Future validation studies should examine whether the distinction between input-related privacy and data concerns and output-related reliability concerns holds across generative AI systems, tasks, and learning contexts.

The gender composition and recruitment design also constrain generalizability. Female participants constituted 74.8% of the sample, and scalar invariance was not established; latent-mean and structural comparisons across gender could therefore not be conducted, leaving the applicability of the structural findings to male first-year students insufficiently established. Participants were recruited through a non-probability, voluntary-response procedure at six public universities in central China, introducing potential self-selection bias and restricting the sampling frame to this regional context; because institutional identifiers were not retained, within-institution dependence and between-university variation could not be examined. Future studies should recruit more gender-balanced samples, preserve institutional information, and replicate the model across public and private institutions, regions, educational stages, disciplines, and recruitment channels. Such designs would permit institution-level variation to be modeled and support tests of measurement invariance across institutional and cultural settings.

Future research should identify the conditions under which risk concerns become relevant to use decisions. Preregistered studies could examine individual characteristics such as prior AI experience, AI and privacy literacy, verification skills, and self-efficacy; appraisals such as trust, enjoyment, and perceived usefulness; and contextual conditions such as task stakes and institutional safeguards. Experimental and longitudinal designs could vary task consequences, access to verification resources, and governance conditions. Evaluations of the ICRM sequence described in Section 5.3 should assess source verification, disclosure, privacy-protective choices, trust calibration, learning performance, and appropriate reliance through platform logs, task records, behavioral choices, and performance measures. These studies would clarify how reported intentions relate to actual patterns of generative AI use.

## 6 Conclusion

This study distinguished utility judgments from privacy and data concerns and output reliability concerns in examining generative AI use intention among first-year university students. Perceived ease of use was associated with use intention through perceived usefulness, while intolerance of uncertainty was consistently associated with stronger concerns in both risk domains. The associations of these concerns with use intention varied across analytical specifications, showing that sensitivity to uncertain risks and the relationship between risk appraisal and use intention are theoretically distinct. Utility judgments emerged as the most stable correlates of use intention, while the separation of the two risk domains points to the value of task-specific guidance for privacy protection, output verification, and human review.

## Data Availability

The raw data supporting the conclusions of this article will be made available by the authors, without undue reservation.
