# Supplementary material for: Utility judgments, risk concerns, and generative AI use intention among first-year university students
Source: Front Psychol. 2026 Sep 21;17:1924148. doi: 10.3389/fpsyg.2026.1924148 (PMC13635203; doi:10.3389/fpsyg.2026.1924148)
Supplement: Supplementary file 1 [file Supplementary_file_1.DOCX]

**Supplementary Material**

All analyses use *N* = 504 unless stated otherwise.

**Supplementary Table S1. Heterotrait–monotrait ratio (HTMT2) among constructs.**

| **Construct** | **PR** | **RR** | **PEOU** | **PU** | **BI** |
| --- | --- | --- | --- | --- | --- |
| PR | — |  |  |  |  |
| RR | .701 | — |  |  |  |
| PEOU | .155 | .317 | — |  |  |
| PU | .185 | .287 | .668 | — |  |
| BI | .053 | .183 | .481 | .755 | — |

*Note.* The lower triangle reports HTMT2 values. HTMT2 was calculated from polychoric item correlations using the geometric-mean implementation in semTools::htmt(). PR = privacy and data concerns; RR = output reliability concerns; PEOU = perceived ease of use; PU = perceived usefulness; BI = generative AI use intention. IU was not included because it was modeled with a bifactor structure.

**Supplementary Table S2. Fornell–Larcker discriminant validity matrix.**

| **Construct** | **PR** | **RR** | **PEOU** | **PU** | **BI** |
| --- | --- | --- | --- | --- | --- |
| PR | **.831** |  |  |  |  |
| RR | .733 | **.797** |  |  |  |
| PEOU | .172 | .316 | **.729** |  |  |
| PU | .207 | .303 | .695 | **.852** |  |
| BI | .083 | .206 | .524 | .767 | **.781** |

*Note.* Bold diagonal values are √AVE; the lower triangle contains latent correlations from the final integrated measurement model. PR = privacy and data concerns; RR = output reliability concerns; PEOU = perceived ease of use; PU = perceived usefulness; BI = generative AI use intention. IU was not included because it was modeled with a bifactor structure.

**Supplementary Table S3. Structural estimates across three analytical settings (*N* = 504).**

*Panel A. M2a, the formal hypothesis-testing model*

| **Path / index** | **Composite-score MLR (primary)** | **Latent MLR** | **Ordered-item WLSMV** |
| --- | --- | --- | --- |
| PEOU → PU (β) | .507*** | .645*** | .727*** |
| PU → BI (β) | .602*** | .759*** | .751*** |
| IU → PR (β) | .300*** | .309*** | .375*** |
| IU → RR (β) | .308*** | .340*** | .453*** |
| PR → BI (β) | −.053 (*p* = .228) | −.110 (*p* = .119) | −.196 (*p* = .002) |
| RR → BI (β) | .028 (*p* = .579) | .085 (*p* = .281) | .289 (*p* < .001) |
| Indirect PEOU → PU → BI | *B* = 0.286, 95% CI [0.210, 0.370] | β = .490 (*p* < .001) | β = .546 (*p* < .001) |
| Indirect IU → PR → BI | *B* = −0.017, 95% CI [−0.043, 0.011] | β = −.034 (*p* = .107) | β = −.074 (*p* = .002) |
| Indirect IU → RR → BI | *B* = 0.009, 95% CI [−0.022, 0.045] | β = .029 (*p* = .316) | β = .131 (*p* < .001) |
| Combined indirect IU → PR/RR → BI | *B* = −0.008, 95% CI [−0.035, 0.025] | β = −.005 (*p* = .786) | β = .057 (*p* = .001) |
| χ² (*df*) | 36.69 (7) | 630.907 (412) | 1250.898 (412) |
| CFI | .932 | .953 | .949 |
| TLI | .855 | .947 | .943 |
| RMSEA [90% CI] | .092 [.067, .118] | .037 [.031, .043] | .064 [.060, .068] |
| SRMR | .073 | .065 | .074 |

*Panel B. M2b, the exploratory extension adding IU → BI*

| **Path / index** | **Composite-score MLR (primary)** | **Latent MLR** | **Ordered-item WLSMV** |
| --- | --- | --- | --- |
| IU → BI (β) | .166 (*p* = .001) | .183 (*p* = .004) | .100 (*p* = .058) |
| M2b vs. M2a, scaled Δχ² (1) | 12.015 (*p* < .001) | 2.885 (*p* = .089) | 3.288 (*p* = .070) |
| χ² (*df*) | 21.43 (6) | 624.000 (411) | 1261.686 (411) |
| CFI / TLI | .965 / .912 | .955 / .949 | .949 / .942 |
| RMSEA [90% CI] | .071 [.044, .101] | .037 [.031, .042] | .064 [.060, .068] |
| SRMR | .063 | .063 | .073 |

*Panel C. WLSMV diagnostic specifications for the two risk paths*

| **Diagnostic specification (WLSMV)** | **Estimate** | ***p*** |
| --- | --- | --- |
| PR → BI, RR → BI path omitted | β = .047 | .166 |
| RR → BI, PR → BI path omitted | β = .147 | < .001 |
| PR–RR residual correlation, joint model | .686 | — |

*Note.* The composite-score MLR column is the primary analysis; both latent columns use the same structural paths and residual-covariance specification as the primary analysis (IU correlated with PEOU; PR and RR residuals correlated). Indirect associations in the composite-score column are unstandardized and were obtained from 5,000 nonparametric bootstrap resamples with percentile 95% confidence intervals. Coefficients in the WLSMV column are estimated from polychoric correlations and are therefore not directly comparable in magnitude with the two maximum-likelihood columns. For latent MLR, CFI, TLI, and RMSEA are robust indices. For ordered-item WLSMV, the reported χ², CFI, TLI, and RMSEA values are WLSMV-scaled indices, consistent with the WLSMV measurement-model reporting in the main manuscript. The composite-score and latent MLR nested comparisons used Satorra–Bentler scaled χ² difference tests; the ordered-item WLSMV comparison used lavaan’s scaled χ² difference procedure. M2b is exploratory and does not enter the determination of H1–H6. ****p* < .001.

**Supplementary Table S4. IU dimension sensitivity analyses (*N* = 504).**

| **Model** | **IU → PR (β)** | **IU → RR (β)** | **Direct IU → BI (exploratory)** | **Indirect via PR, *B* [95% CI]** | **Indirect via RR, *B* [95% CI]** | **Model fit** |
| --- | --- | --- | --- | --- | --- | --- |
| Prospective IU only | .269*** | .281*** | β = .145 (*p* = .002) | −0.015 [−0.039, 0.010] | 0.008 [−0.020, 0.041] | χ²(7) = 33.742; CFI = .948; TLI = .889; RMSEA = .099 [.067, .134]; SRMR = .072 |
| Inhibitory IU only | .280*** | .282*** | β = .153 (*p* = .004) | −0.012 [−0.032, 0.010] | 0.007 [−0.017, 0.034] | χ²(7) = 36.428; CFI = .942; TLI = .876; RMSEA = .104 [.072, .139]; SRMR = .074 |
| Both dimensions | P = .148*  I = .181** | P = .167**  I = .170** | P: β = .078 (*p* = .109)  I: β = .104 (*p* = .085) | P: −0.008 [−0.025, 0.006]; I: −0.008 [−0.024, 0.006] | P: 0.005 [−0.013, 0.028]; I: 0.004 [−0.011, 0.023] | χ²(9) = 38.907; CFI = .961; TLI = .909; RMSEA = .091 [.063, .122]; SRMR = .070 |

*Note.* P = prospective IU; I = inhibitory IU. Each dimension was first entered separately and then both were entered simultaneously, using the same structural specification as the primary composite-score model. Model-fit indices refer to the corresponding specification without the exploratory direct path(s) from the IU dimension(s) to BI; coefficients in the “Direct IU → BI” column were obtained from separate exploratory extensions. Direct paths from either dimension to BI are exploratory and do not enter the determination of H1–H6. All 5,000-resample percentile bootstrap confidence intervals for the indirect associations included zero. **p* < .05. ***p* < .01. ****p* < .001.

**Supplementary Table S5. Gender measurement-invariance sensitivity analyses (*N* = 504; 127 male, 377 female).**

| **Model / step** | **Estimator** | **χ² (*df*)** | **Robust CFI** | **Robust TLI** | **Robust RMSEA [90% CI]** | **SRMR** | **ΔCFI** | **ΔRMSEA** | **ΔSRMR** | **Decision** |
| --- | --- | --- | --- | --- | --- | --- | --- | --- | --- | --- |
| Ordinal configural | WLSMV | — | — | — | — | — | — | — | — | Not estimable; six item-by-gender combinations contained an empty response category |
| Configural | MLR | 1179.516 (810) | .934 | .924 | .046 [.040, .052] | .059 | — | — | — | Admissible |
| Loading (metric) | MLR | 1239.771 (845) | .930 | .923 | .046 [.041, .052] | .063 | −.004 | < .001 | .004 | Supported |
| Scalar | MLR | 1270.642 (868) | .928 | .922 | .047 [.041, .052] | .064 | −.002 | < .001 | .001 | Inadmissible; negative residual variance for iu2 in the male group |

*Note.* Multigroup WLSMV was selected first because the indicators use five ordered response categories. Response categories were not combined post hoc, because repeated merging would change the original measurement scale and introduce data-dependent scoring decisions. The MLR analyses treat the five-category indicators as approximately continuous and are reported as a supplementary feasibility check. Δ values compare each model with the preceding one. The analysis supports configural and loading invariance only; no latent-mean or structural comparison across gender is claimed, and the generalizability limitation arising from the gender imbalance remains. Fit and change indices for the scalar model are reported for completeness; because the solution was inadmissible, they are not interpretable.

**Supplementary Table S6. Construct labels and analytical representations.**

| **Abbreviation** | **Construct name** | **Usage in narrative text** | **Analytical representation** |
| --- | --- | --- | --- |
| PEOU | Perceived ease of use | PEOU | Four-item mean score in the primary models; latent factor in sensitivity models |
| PU | Perceived usefulness | PU | Four-item mean score in the primary models; latent factor in sensitivity models |
| BI | Generative AI use intention | use intention | Three-item mean score in the primary models; latent factor in sensitivity models |
| PR | Privacy and data concerns | PR | Four-item mean score in the primary models; latent factor in sensitivity models |
| RR | Output reliability concerns | RR | Four-item mean score in the primary models; latent factor in sensitivity models |
| IU | Intolerance of uncertainty | IU | Twelve-item overall mean score in the primary models; general IU factor in latent bifactor models |
| Prospective IU | Prospective intolerance of uncertainty | Prospective IU | Seven-item mean score in dimension-level sensitivity analyses |
| Inhibitory IU | Inhibitory intolerance of uncertainty | Inhibitory IU | Five-item mean score in dimension-level sensitivity analyses |

*Note.* BI is labeled as use intention in narrative text and as BI in hypotheses, figures, tables, and model notation. Overall IU refers to the 12-item composite score used in the primary path models, whereas the general IU factor refers to the general latent factor estimated in the bifactor measurement and latent structural models.
